# *In vivo *killing of *Staphylococcus aureus *using a light-activated antimicrobial agent

**DOI:** 10.1186/1471-2180-9-27

**Published:** 2009-02-04

**Authors:** Parjam S Zolfaghari, Samantha Packer, Mervyn Singer, Sean P Nair, Jon Bennett, Cale Street, Michael Wilson

**Affiliations:** 1 Bloomsbury Institute of Intensive Care Medicine, Wolfson Institute for Biomedical Research, University College London, Gower Street, London, WC1E 6BT, UK; 2 Division of Microbial Disease, UCL Eastman Dental Institute, University College London, 256 Grays Inn Road, London, WC1X 8LD, UK; 3 Peninsula College of Medicine and Dentistry, John Bull Building, Tamar Science Park, Plymouth, PL6 8BU, UK; 4 Ondine Biopharma Corporation, 910-1100 Melville Street, Vancouver, BC, V6E 4A6, Canada

## Abstract

**Background:**

The widespread problem of antibiotic resistance in pathogens such as *Staphylococcus aureus *has prompted the search for new antimicrobial approaches. In this study we report for the first time the use of a light-activated antimicrobial agent, methylene blue, to kill an epidemic methicillin-resistant *Staphylococcus aureus *(EMRSA-16) strain in two mouse wound models.

**Results:**

Following irradiation of wounds with 360 J/cm^2 ^of laser light (670 nm) in the presence of 100 μg/ml of methylene blue, a 25-fold reduction in the number of viable EMRSA was seen. This was independent of the increase in temperature of the wounds associated with the treatment. Histological examination of the wounds revealed no difference between the photodynamic therapy (PDT)-treated wounds and the untreated wounds, all of which showed the same degree of inflammatory infiltration at 24 hours.

**Conclusion:**

The results of this study demonstrate that PDT is effective at reducing the total number of viable EMRSA in a wound. This approach has promise as a means of treating wound infections caused by antibiotic-resistant microbes as well as for the elimination of such organisms from carriage sites.

## Background

The emergence of resistant strains of bacteria such as methicillin-resistant *Staphylococcus aureus *(MRSA) poses a major challenge to healthcare. MRSA is a major cause of hospital-acquired infection throughout the world and is now also prevalent in the community as well as nursing and residential homes [1-3]. Of the *Staph. aureus *isolates in the United Kingdom in 2005, 43.6% were found to be MRSA and a point prevalence survey showed that 16% of intensive care patients were either colonized or infected with MRSA [4,5]. Mortality attributable to MRSA bacteraemia has been estimated to be 22% [6]. Increasing reports of resistance to antibiotics and antiseptics, have sparked a wave of research to find alternative antimicrobial strategies [7,8]. One such strategy involves the use of light-activated antimicrobial agents (LAAAs) in photodynamic therapy (PDT) [9]. Following excitation of the LAAA by light of an appropriate wavelength, singlet oxygen and free radicals are generated locally which directly attack the plasma membrane and other cellular targets resulting in bacteriolysis [10,11]. This could form the basis of an alternative approach for the eradication of such bacteria from superficial wounds, burns, varicose ulcers, pressure sores and carriage sites which are readily accessible to topical application of a LAAA and light.

*In vitro *experiments with PDT have demonstrated effective bactericidal activity of toluidine blue O (TBO) and methylene blue (MB) as photosensitisers against MRSA [12-14]. However, there are few *in vivo *studies which have looked at the effect of PDT in wounds, and in particular ones inoculated with drug-resistant bacteria. Furthermore there are no reports of the use of PDT in wounds colonised by MRSA. Two mouse studies that investigated the effect of PDT using a targeted polycationic photosensitiser demonstrated that PDT is effective at reducing the number of bacteria in excision wounds infected with *Escherichia coli *and *Pseudomonas aeruginosa *[15,16]. This was also shown in a burn wound model infected with bioluminescent *Staphylococcus aureus *treated with PDT using a cationic porphyrin [17]. However, within days of treatment, the bacterial luminescence reappeared, indicating incomplete bacterial killing.

A potential problem with PDT however, is its lack of specificity. Its cytotoxic effect, which destroys bacteria so effectively, leads to delayed burn-wound healing, presumably as a result of the reactive oxygen species acting on host tissue [17]. PDT also resulted in delayed healing of wounds in rat skin grafts [18]. However, treatment of wounds with laser light alone shows more diverse findings. Delayed wound healing was seen after delivery of high laser energy (211–420 J/cm^2^) in burn wounds [17] in contrast to unchanged or even improved speed of recovery when lower light energy (upto 75 J/cm^2^) is used [18,19].

A further factor associated with red light illumination is the generation of heat. This is partly due to absorption of light by endogenous chromophores as well as release of energy by the excited photosensitiser in the form of heat rather than the actual PDT effect. As far as we are aware, no *in vivo *study has investigated the local heating effect associated with PDT treatment for microbial eradication using methylene blue.

The aims of this study were to evaluate the effect of PDT, using methylene blue as a photosensitiser, on the survival of an epidemic strain of MRSA in excisional and superficial wounds in mice. The local heating effect associated with this PDT treatment was evaluated as well as the extent of collateral damage to host tissue.

## Results

### Effect of PDT on the number of viable bacteria in the wounds

**Figure 1:**
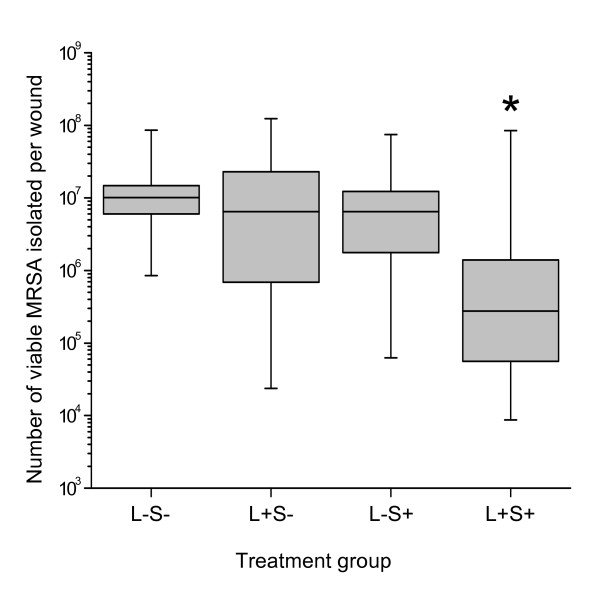
**Box- and whisker plot of the number of viable MRSA isolated from excision wounds treated with photodynamic therapy (PDT)**. The wounds were inoculated with EMRSA-16 for one hour, treated with PDT using methylene blue and 665 nm laser light (360 J/cm^2^) and examined immediately after treatment. A 25 fold reduction in the number of viable MRSA was seen in the PDT wounds (L+S+) compared to the controls. Results are presented as box (median, 25^th ^and 75^th ^centiles) and whiskers (minimum and maximum values), n = 12 per group (* indicates p < 0.008).

**Figure 2:**
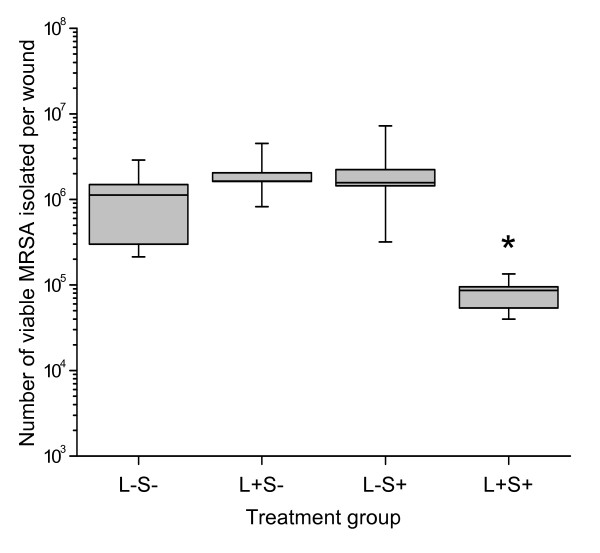
**Box- and whisker plot of the number of viable MRSA isolated from superficial scarified wounds following photodynamic therapy**. The wounds were examined immediately after treatment. A 14-fold reduction in the number of viable bacteria was observed in the PDT treated wounds (L+S+) compared to the control wounds. (* indicates p = 0.002).

Figures 1 and 2 show the number of EMRSA-16 isolated from the treated excision and superficial wounds and their respective control groups (wounds that did not receive any treatment, wounds that did not receive MB, and those that were not irradiated).

Irradiation of the wounds in the presence of MB resulted in a significant reduction in the number of viable bacteria recovered from the wounds. This reduction was 25 fold (1.40 log_10 _CFU/wound) in the excision wounds and 14 fold (1.15 log_10 _CFU/wound) in the superficial scarified wounds.

### Effect of PDT on the temperature of the wounds

**Figure 3:**
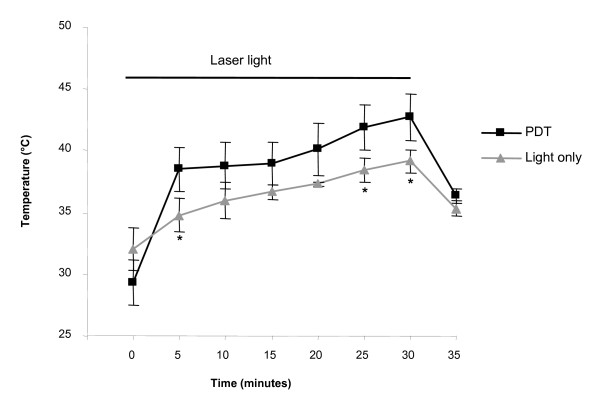
**Effect of laser light alone and laser light with methylene blue on wound temperature**. Temperature was measured using a thermistor tunnelled into the centre of the wounds. There was an immediate increase in the temperature of the wounds following the start of irradiation with laser light of 665 nm wavelength and power rating of 200 mW/cm^2^. There was a bigger increase in temperature in the PDT treated wounds (black squares) than in the light only (grey triangles) treated group. The temperature dropped upon cessation of irradiation.

To study the effects of irradiation on wound temperature, two groups of animals were examined. One group received only laser irradiation with no MB (L+S-; n = 3) while the other group had full PDT treatment with MB and laser irradiation (L+S+; n = 3). The wound temperatures at the beginning of treatment were consistently lower than the core temperatures. The wound temperature in the animals treated with PDT rose by 13.4 ± 0.5°C and the maximum temperature achieved in this group was 44.5°C (Figure 3). However, a smaller increase in temperature was noted in wounds irradiated with laser light in the absence of MB (7.1 ± 2.6°C) with 40.1°C being the highest temperature reached in this group.

### Histological findings following PDT

The cytotoxic effect of PDT on host tissue was examined in 18 biopsies from wounds treated with laser light and MB in combination. All exhibited a clear demarcation between wound and the skin and extended into adipose or loose areolar tissue on their deep aspect. Some included fragments of the underlying skeletal muscle. In the area of the wound, the epidermis had been removed to leave either a thin layer of the underlying connective tissue overlying the panniculus adiposus, or a wound base of adipose tissue. In contrast, the adjacent tissue had retained its epidermis complete with appendages. None of the wounds examined showed evidence of extensive tissue necrosis.

**Figure 4:**
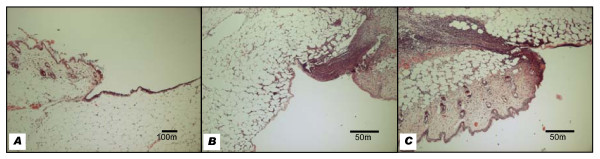
**Haematoxylin & Eosin stained sections of treated and control wounds**. (A) Normal tissue architecture is seen in wounds taken immediately after treatment with photodynamic therapy. (B) At 24 hours, a dense cellular infiltrate appears at the wound edges inoculated with MRSA and treated with methylene blue only (L-S+). There are also smaller, focal lymphocytic aggregates infiltrating between subcutaneous adipocytes in the wound crater. (C) Following photodynamic therapy with laser light and methylene blue (L+S+), the wounds show a dense cellular infiltrate at the edges and the subcutaneous fat very similar to the control wounds.

Normal wound architecture was seen in wounds that were sampled immediately after PDT (Figure 4A). By 24 hours there was a heavy lymphocytic infiltrate, which in some sections extended quite deeply to involve the underlying muscle. This was very prominent at the wound edges but less marked towards the centre (Figure 4B). When present in the latter areas, inflammatory cells could be seen infiltrating between dermal adipocytes. Wounds examined at 24 hours in the presence of bacteria exhibited a similar pattern of inflammatory cell infiltration regardless of whether they were treated with laser light and MB, either alone or in combination (Figure 4C). Moderate to heavy bacterial deposits were observed in some wounds and were generally localised to areas with a heavy fibrin slough. Observations were made on three biopsies for each experimental condition.

## Discussion

There are many reports in the literature of the ability of light-activated antimicrobial agents to kill a wide range of microbes in the laboratory [9,20]. In some of these *in vitro *investigations, attempts have been made to model the *in vivo *situation by using biofilms of the target organisms [21] or by carrying out experiments in the presence of blood or serum.[22,23] In this study we have taken this further by investigating the ability of a LAAA, methylene blue, to kill bacteria while present in a wound. Our *in vivo *model reflects the early stages of an infectious process i.e. the initial colonisation of a wound by a potential disease-inducing organism. We used a strain of MRSA that is known to cause wound infections with significant clinical relevance, including fatal outcomes. The results of our study demonstrate for the first time that it is possible to reduce the number of viable MRSA present in a wound using the LAAA methylene blue when activated by 360 J/cm^2 ^of light (with a wavelength of 665 nm – the absorbance maximum of methylene blue) from a low power laser.

Although substantial reductions in the viable count of MRSA in the wounds were achieved, the kills observed in this *in vivo *model were substantially lower than those reported in *in vitro *studies. Hence, using light doses as low as 43 J/cm^2^, 4.7 log_10 _reductions in the viable count of a suspension of MRSA (10^10 ^CFU/ml) were obtained using the LAAA toluidine blue O (a phenothiazinium dye closely related to methylene blue) at a concentration of 12.5 μg/ml [12]. Wainwright et al. also reported that methylene blue and toluidine blue O are extremely effective LAAAs against MRSA *in vitro *[13]. To our knowledge, only three papers have been published on the use of LAAAs to kill *S. aureus in vivo *[17,24,25]. Each of these has used a different animal model and a different LAAA which makes comparisons with the present study difficult. However, in all of these studies the bacterial kills reported were considerably lower than those that can be achieved *in vitro*. For example, when the LAAA meso-mono-phenyl-tri(N-methyl-4-pyridyl)-porphyrin (PTMPP) was used to kill *S. aureus *in burn wounds in mice, the kills achieved amounted to less than 2 log_10 _units using a light dose of 211 J/cm^2 ^[17]. Much greater kills were attained *in vitro *using a considerably lower light dose (0.6 J/cm^2 ^compared with 211 J/cm^2^) and concentration of PTMPP (1.6 μM *in vitro *compared with 500 μM *in vivo*). Several factors may account for the reduced bactericidal effect observed *in vivo *including: (i) binding of the LAAA to host material thereby reducing the effective dose and resulting in the generation of singlet oxygen in regions remote from the target bacteria, (ii) absorption of laser light by LAAA bound to host tissues – this would result in shielding of any LAAA bound to bacteria preventing light activation and (iii) quenching of singlet oxygen by host molecules thereby protecting bacteria from its harmful effects. Some of these problems could be avoided, and hence greater kills achieved *in vivo*, by using a photosensitiser covalently linked to a bacterial targeting moiety [15,24].

One aspect of the *in vivo *use of antimicrobial PDT that has not previously been investigated is the change in temperature of the host tissues accompanying the procedure. Treatment of basal cell carcinoma with 5-aminolevulinic acid and red light (590–700 nm) with a power density of 100 mW/cm^2 ^resulted in a 8–10°C change in the surface temperature of the lesion [26]. In our study we found that irradiation with 360 J/cm^2 ^of light in the presence of methylene blue resulted in a substantial rise in the wound temperature – the average maximum temperature at the centre of the wounds being 42.7 ± 1.8°C. However, it is very unlikely that such a temperature increase could account for the bacterial kills observed – *S. aureus *is able to grow at temperatures as high as 45°C [27]. Furthermore, the decimal reduction time for the organism at a higher temperature of 50°C is of the order of 105 minutes whereas in the current study, the wound temperature was above 40°C for no longer than 10 minutes and did not reach 45°C [28].

Microscopic examination of biopsies immediately following treatment and after 24 hours did not reveal any tissue necrosis regardless of the experimental treatment applied. Thus, at the 24 hour time point the use of PDT did not amplify the effect of the wounding.

This study has demonstrated that substantial kills of MRSA can be achieved in an *in vivo *mouse wound model using the LAAA methylene blue, and without causing collateral damage to host tissues. These findings are significant for several reasons. They constitute the first report of the *in vivo *killing of MRSA using LAAAs. Secondly, they support the small, but growing, number of *in vivo *studies demonstrating that PDT is an effective antimicrobial. Thirdly, if such results can be reproduced in humans, the technique could be an effective means of preventing the colonisation of wounds by the organism and, possibly be used to eliminate MRSA from carriage sites such as the anterior nares. It should be noted that only a single application of PDT was used in this study and greater kills may be achieved through repeated application of the technique or by the "fractionation" of the light dose administered or in combination with other therapeutic agents such as antibiotics. We are currently investigating such modifications of the technique.

In this era of concern over the threat of the growing antibiotic resistance of pathogens such as MRSA to antibiotics, PDT offers an important advantage in that it is unlikely that the organism could develop resistance to this modality[11,14]. Another advantage of PDT is that, unlike the vast majority of antibiotics, it can also inactivate microbial virulence factors in addition to its microbicidal effect. Hence, the biological activities of the proteases of *Pseudomonas aeruginosa *and *Porphyromonas gingivalis *and the lipopolysaccharide of *Escherichia coli *have all been shown to be reduced by irradiation in the presence of a LAAA [29,30]. The future of LAAAs for the prevention and/or treatment of infectious diseases looks promising following the recent report of the use of methylene blue to successfully treat periodontitis – one of the most prevalent infectious diseases of humans.[31]

## Conclusion

In this study we have shown that PDT using the light-activated antimicrobial agent, methylene blue, kills MRSA in superficial and deep excisional wounds in mice. However, killing is less effective than when performed *in-vitro*. This bactericidal effect was not due to the heat generated as a consequence of the treatment. Histological examination of the wounds showed neither collateral tissue necrosis nor architectural disturbance.

## Methods

### Bacteria

The organism used in this investigation was the prototypic UK epidemic MRSA: EMRSA-16 (NCTC 13143). EMRSA-16 was maintained by weekly sub-culture on blood agar (BA, Oxoid Ltd, Basingstoke, UK) supplemented with 5% (v/v) horse blood. For experimental purposes, a few colonies were inoculated into brain heart infusion broth (BA, Oxoid Ltd, Basingstoke, UK) and grown aerobically with shaking for 16 hours at 37°C. Cells were then harvested by centrifugation, washed and resuspended in sterile phosphate buffered saline (PBS) to a concentration of 4 × 10^9 ^bacteria per ml. Twenty five μl of the bacterial suspension (10^8 ^CFU of EMRSA-16) was then added to the wound.

### Photosensitiser and laser

Methylene blue (MB, Sigma, UK) solution was prepared fresh for each experiment in sterile PBS to a final concentration of 100 μg/ml. The light source used was a 665 nm diode laser (PerioWave system, Ondine Biopharma, Vancouver, Canada) with a measured output of 200 mW distributed by a fibreoptic cable and a diffusing head. The source was held at a constant distance from the wound to produce a 1 cm^2 ^circle of illumination.

### Animals

All animal experiments were carried out in accordance with the Animals (Scientific Procedures) Act 1986 and with approval of the local Ethics Committee. Eight-week old female C57 Black mice (Charles River, Margate, Kent, UK), of 14–18 g body weight were housed in the local animal unit for 7 days prior to experimentation, with free access to food and water.

### Excisional wound model

Mice were anaesthetised with an intramuscular injection of ketamine-xylazine mixture (90 mg/kg ketamine, 9 mg/kg xylazine), and their backs shaved and depilated with a commercial cream (Veet^®^, Reckitt Benckiser, UK). Intramuscular Carpofen (5 mg/kg) was used to provide analgesia. At 45 minutes post-inoculation, the mice received a second dose of the anaesthetic mixture to allow for the subsequent treatment. The skin was washed with 70% (v/v) ethanol and left to dry prior to wound creation. Excision wounds were created by pinching and lifting the skin of the back using sterile forceps and cutting a 6 mm circular (28 mm^2^) area using sharp scissors to cut down to the subcutaneous areolar tissue. Twenty-five μl of the bacterial suspension was then added to the wound (10^8 ^CFU of EMRSA-16), and incubated for one hour prior to treatment. MRSA was found to be the predominant bacterium colonising the wound at day 5 (data not included).

### Superficial wound model

The preparation of the animals for this model was as described for the excisional wound model above. 25 mm^2 ^square shaped wounds were created in the skin of the back by scarification using a 27G needle, run ten times parallel in one direction and another ten times perpendicular to the original tracks. The wounds were visibly red and mildly swollen after 30 minutes. Ten μl of the bacterial suspension was placed on the wound (4 × 10^7 ^CFU of EMRSA-16), and incubated for one hour prior to treatment. This method also resulted in a reproducible MRSA wound colonisation model, which persisted for up to 5 days post inoculation (data not shown).

### Photodynamic therapy (PDT)

All experiments were carried out under subdued room lighting. PDT was performed 1 hour after inoculating the wounds with the bacterial suspension. The excision wounds received 25 μl of MB (100 μg/ml) solely at the start of irradiation, whilst the superficial scarified wounds received 10 μl of MB just before the start of irradiation and a further 10 μl after 15 minutes of irradiation. The wounds were irradiated immediately after the application of MB and continued for 30 minutes. This equated to a total delivered light dose of 360 J/cm^2^. Following the completion of treatment, a circular area of skin and associated subcutaneous tissue of 1 cm diameter with the wound at its centre, was removed using sterile scissors. These were then placed in 0·5 ml Stuart's transport medium and shielded from light until delivery to the microbiology laboratory for processing and analysis within 2 hours. The animals were subsequently culled in accordance with the Animal Scientific Procedures act (1986).

Control groups were used to test the effect of MB alone (by incubating wounds in the dark for the equivalent time period as needed for irradiation, L-S+, where L denotes light treatment and S denotes photosensitiser), light alone (by illuminating wounds in the absence of MB, L+S-). A final untreated control group received no MB or light illumination (L-S-). PBS was used instead of MB in the control wounds that received no MB.

Twelve mice per group were examined in the excision wound model, whereas 6 mice per group were used in the superficial scarified wound model.

In preliminary experiments, the dose of MB (concentration and volume of solution) was optimised to achieve maximum bacterial kill. For the excision wounds, 25 μl of MB at a concentration of 100 μg/ml was most effective. However, for the superficial scarified wounds, the same concentration of MB was used but in a reduced volume of 10 μl administered at two separate time-points, 15 minutes apart. The delivered light dose which produced the greatest bacterial kill in both types of wounds was optimised to 360 J/cm^2^, although light doses of 180 J/cm^2 ^also reduced the number of viable bacteria recovered.

### Processing of tissue samples

Using a micro-Eppendorf pestle, the tissue in Stuart's transport medium was minced to release the bacteria within the wound. Tissue samples treated with MB were kept in the dark during processing. The contents of the Eppendorf tube were transferred into 4.5 ml of PBS. Aliquots of serial 10-fold dilutions of the suspension were plated onto half plates of BA and mannitol salt agar (MSA). Plates were incubated at 37°C in air for 36 hours before colonies of EMRSA-16 were counted. Results represent the mean CFU of EMRSA-16 recovered per wound based on counts from both BA and MSA plates for each sample.

### Histological evaluation

For these studies, wounds were removed either immediately or after 24 hours following treatment and fixed in 4% formal saline for 24 hours. The specimens were processed and embedded in paraffin wax. 6 μm histological sections were cut stained with haematoxylin-eosin and examined by light microscopy.

### Wound temperature studies

Following creation and inoculation of the excision wounds with bacteria for 1 hour, a 1 mm diameter thermistor (Thermilinear^® ^component, Yellow Spring Instruments Co., Ohio, USA) was tunnelled subcutaneously from an entry point 2 cm away from the wound to its centre, avoiding disruption of the wound integrity. PDT was then performed as above and temperature changes plotted. A single control group had wounds irradiated with laser light in the absence of MB (L+S-).

### Statistical analysis

Data are expressed as mean ± standard error or median (95% confidence intervals). Group comparison for continuous variables was tested with the t-test (for temperature changes) and Mann Whitney U test for the rest of the data. Multiple comparisons increase the risk of type I errors. In order to prevent such errors, we used the Bonferroni method and divided the 5% alpha level by the number of comparisons. Hence, when pair-wise comparisons were performed between treatment groups, p was only significant if it was < 0.008. All tests were performed with the use of SPSS 14.0 for Windows.

## Abbreviations

**EMRSA**: Epidemic methicillin-resistant *Staph. aureus*; **LAAA**: Light-activated antimicrobial agent; **L+S+**: Wounds treated with both methylene blue and laser light; **L+S-**: Control wounds treated with laser light only; **L-S+**: Control wounds treated with methylene blue only; **L-S-**: Control wounds with no treatment; **MB**: Methylene blue; **MRSA**: Methicillin-resistant *Staph. Aureus*; **PDT**: Photodynamic therapy; **TBO**: Toluidine blue O; **CFU**: Colony forming units.

## Competing interests

MW is a member of the Scientific Advisory Board of Ondine Biopharma Inc. and holds shares in this company, PSZ received financial income from Ondine Biopharma Inc. during the course of the study. CS is director of research at Ondine Biopharma Inc. Other authors: None to declare.

## Authors' contributions

PSZ carried out all the animal experiments including all photodynamic therapy, drafted the manuscript and performed the statistical analysis. SP carried out all microbiological work and analysis and helped draft the manuscript. MS participated in the design of the study and helped drafting the manuscript. JB carried out histological examination of the wounds and helped to draft the manuscript. SPN and MW conceived the study, and participated in its design and coordination and helped to draft the manuscript. CS participated in the design of the study. All authors read and approved the final manuscript.
